# Impact of Article Language in Multi-Language Medical Journals - a Bibliometric Analysis of Self-Citations and Impact Factor

**DOI:** 10.1371/journal.pone.0076816

**Published:** 2013-10-17

**Authors:** Torsten Diekhoff, Peter Schlattmann, Marc Dewey

**Affiliations:** 1 Department of Radiology, Charité - Universitätsmedizin Berlin Campus Mitte, Humboldt-Universität zu Berlin, Freie Universität Berlin, Berlin, Germany; 2 Department of Biometry and Clinical Epidemiology, Charité - Universitätsmedizin Berlin Campus Benjamin-Franklin, Humboldt-Universität zu Berlin, Freie Universität Berlin, Berlin, Germany; 3 Department of Medical Statistics, Computer Sciences and Documentation, University Hospital of Friedrich-Schiller University Jena, Jena, Germany; Max Planck Society, Germany

## Abstract

**Background:**

In times of globalization there is an increasing use of English in the medical literature. The aim of this study was to analyze the influence of English-language articles in multi-language medical journals on their international recognition – as measured by a lower rate of self-citations and higher impact factor (IF).

**Methods and Findings:**

We analyzed publications in multi-language journals in 2008 and 2009 using the Web of Science (WoS) of Thomson Reuters (former Institute of Scientific Information) and PubMed as sources of information. The proportion of English-language articles during the period was compared with both the share of self-citations in the year 2010 and the IF with and without self-citations. Multivariable linear regression analysis was performed to analyze these factors as well as the influence of the journals‘ countries of origin and of the other language(s) used in publications besides English.

We identified 168 multi-language journals that were listed in WoS as well as in PubMed and met our criteria. We found a significant positive correlation of the share of English articles in 2008 and 2009 with the IF calculated without self-citations (Pearson r=0.56, p = <0.0001), a correlation with the overall IF (Pearson r = 0.47, p = <0.0001) and with the cites to years of IF calculation (Pearson r = 0.34, p = <0.0001), and a weak negative correlation with the share of self-citations (Pearson r = -0.2, p = 0.009). The IF without self-citations also correlated with the journal‘s country of origin – North American journals had a higher IF compared to Middle and South American or European journals.

**Conclusion:**

Our findings suggest that a larger share of English articles in multi-language medical journals is associated with greater international recognition. Fewer self-citations were found in multi-language journals with a greater share of original articles in English.

## Introduction

The impact factor (IF) is a measure of an academic journal’s international standing and is influenced by several well-known parameters. Although sometimes harshly criticized, the IF is widely recognized as a quantitative measure of a journal’s reputation and the importance of published items [1]. Therefore, journals attempt to increase their IF in various ways. Some critics believe that journals try to inflate their IF by encouraging self-citations and preferring articles from authors who cite more articles from the journal to which they submit [2]. A study investigating this concern has shown that, at least for anesthesiology journals, there is no broader trend to do so [3].

It is a well-known fact that there is a correlation between a journal’s IF and its language and that it is stronger than the association of IF and country of origin [4,5]. Mueller et al. [4] report that articles written in English are cited more frequently and think it to be imperative that scholars be aware of this language bias, which has also been described by Gregoire et al. [6].

We suggest that a low proportion of self-citations to all citations in a journal is a good indicator of the journal‘s international visibility, especially when compared with the journal’s IF. Therefore, our hypothesis was that multi-language journals with a greater share of English articles have a higher IF without self-citations, on the one hand, and a smaller share of self-citations, on the other hand, as a marker of international recognition. 

## Methods

We analyzed all medical journals that published research manuscripts in English and at least one other language in the years 2008 and 2009. For the analysis we used data from PubMed as well as from WoS. PubMed provided information on the amount of English and non-English language articles as well as on the other language(s) used (query: "2008/01/01"[PDAT]: "2009/12/31"[PDAT]) AND ISSN-No [Journal] AND/NOT English [Language]). WoS provided the total number of cites and self-cites, the number of cites and self-cites to years used in the impact factor (IF) calculation, the overall IF and the IF without self-cites, and the country of origin. 

The first step was to identify the non-English and multi-language journals listed in both PubMed and the WoS Journal Citation Reports. First, we searched PubMed (http://www.ncbi.nlm.nih.gov/pubmed/) for all journals publishing non-English articles in the years specified above (query: ("2008/01/01"[PDAT]: "2009/12/31"[PDAT]) NOT English [Language]). Then we compared the journals retrieved in this way with the 2010 WoS database (http://isiknowledge.com/jcr). The second step was to exclude all journals publishing in their native language only and journals with only nonscientific non-English articles such as conference reports and obituaries. Following these steps, there were only medical journals left that published both non-English and English scientific articles in the years 2008 and 2009 and were listed in both PubMed and WoS. 

In addition, we wanted to ascertain whether or not the journal’s country of origin had any significance. Therefore we divided the territories into five groups: 1) Northern and Western Europe, 2) Eastern and Southern Europe, 3) North America, 4) Middle and South America, and 5) Asia and Africa. 

All data were collected in an Excel table. The statistical analysis was done by an experienced statistician implementing linear regression analysis and Pearson tests for the comparison of the percentage of English articles to impact factor, percentage of self-citations and impact factor without self-citations, respectively. Multifactorial regression analysis and ANOVA test was used to determine the influence of the percentage of English articles, the percentage of self-citations and the number of cites to years used in impact factor calculation on the impact factor without self-citations. We applied a multivariable linear regression analysis to determine the influence of the territory and language used on the results. In order to account for differences between geographic areas those were included as a categorical covariate into the model with Northern and Western Europe as reference category. The used statistic programs were GraphPad Prism v 6.0 and SPSS 20. Statistical significance was assumed for p < 0.05. 

## Results

**Figure 1:**
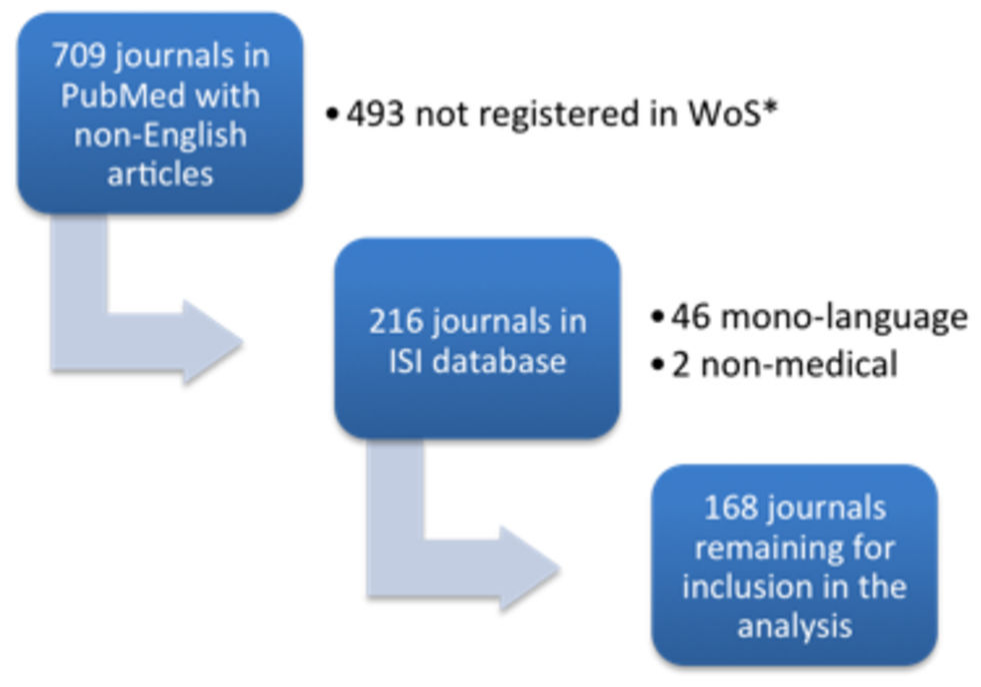
Three-step process of identification of journals and exclusion of journals not meeting our inclusion criteria. *Most journals not registered in WoS were single-language journals from Asia or Eastern Europe.

A total of 127,852 non-English articles were published by 709 journals in 2008 and 2009. Only 216 of these journals were registered in the WoS database (see Figure **1**). After exclusion of all single-language and nonmedical journals registered in WoS and PubMed, 168 journals were left for analysis. Table **1** lists the general characteristics of the 168 journals we investigated. 

**Table 1:** Characteristics of the 168 multi-language medical journals investigated.

	**Overall IF**	**IF without self-cites**	**Cites to years of IF calculation**	**Self-cites to years of IF calculation**	**Proportion (%) of English-language articles**	**Proportion (%) of self-cites**
**Average**	0.86 ± 0.72	0.64 ± 0.52	162 ± 159	38 ± 52	43.2 ± 39	22.8 ± 10
**Minimum**	0.32	0.32	1	0	0.12	0
**Maximum**	4.43	3.0	1091	384	99.8	76.9

**Figure 2:**
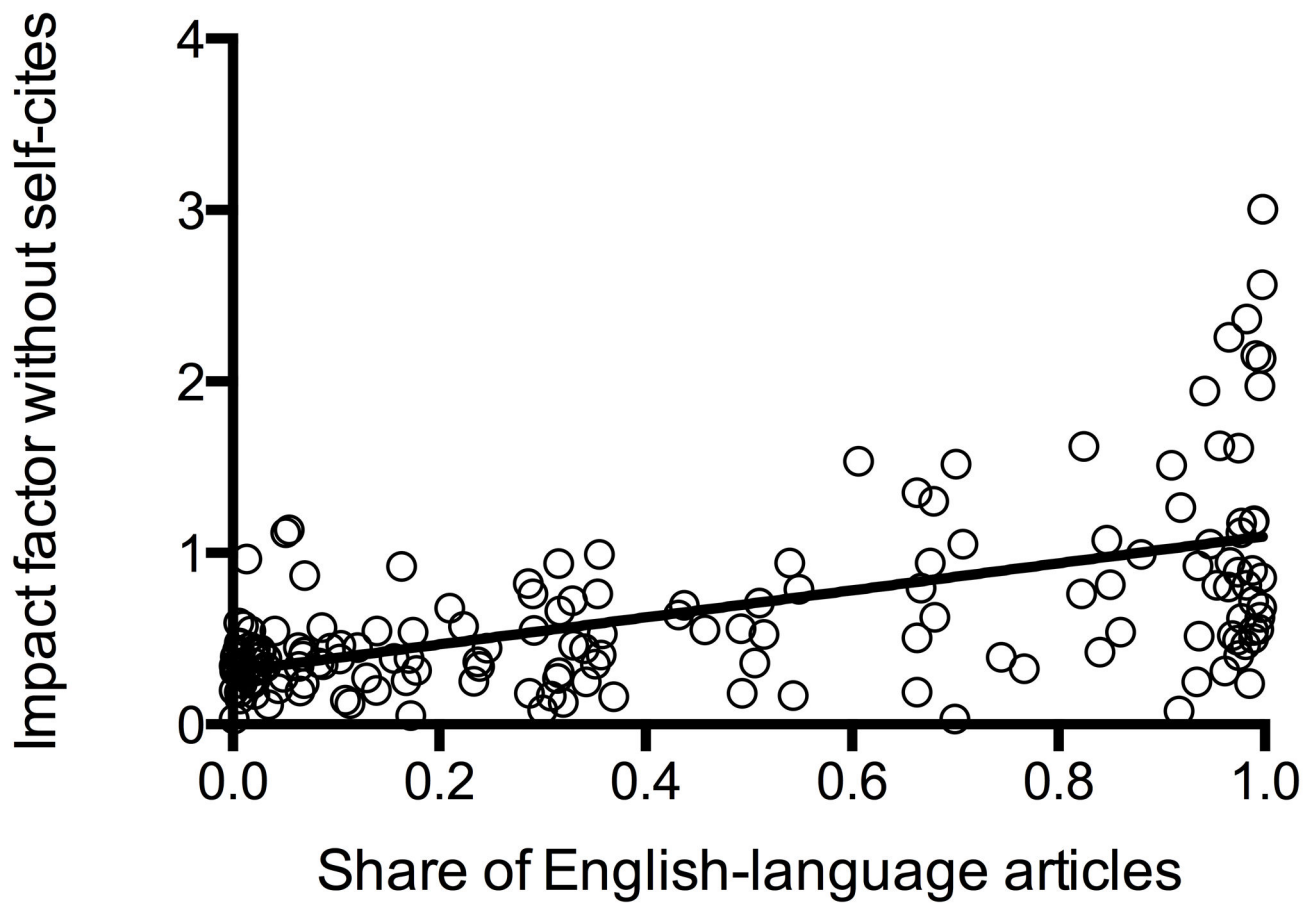
IF without self-citations in relation to the share of English-language articles. There was a moderate correlation: the greater the share of English-language articles, the higher the IF without self-citations (r = 0.56, p = <0.0001).

**Figure 3:**
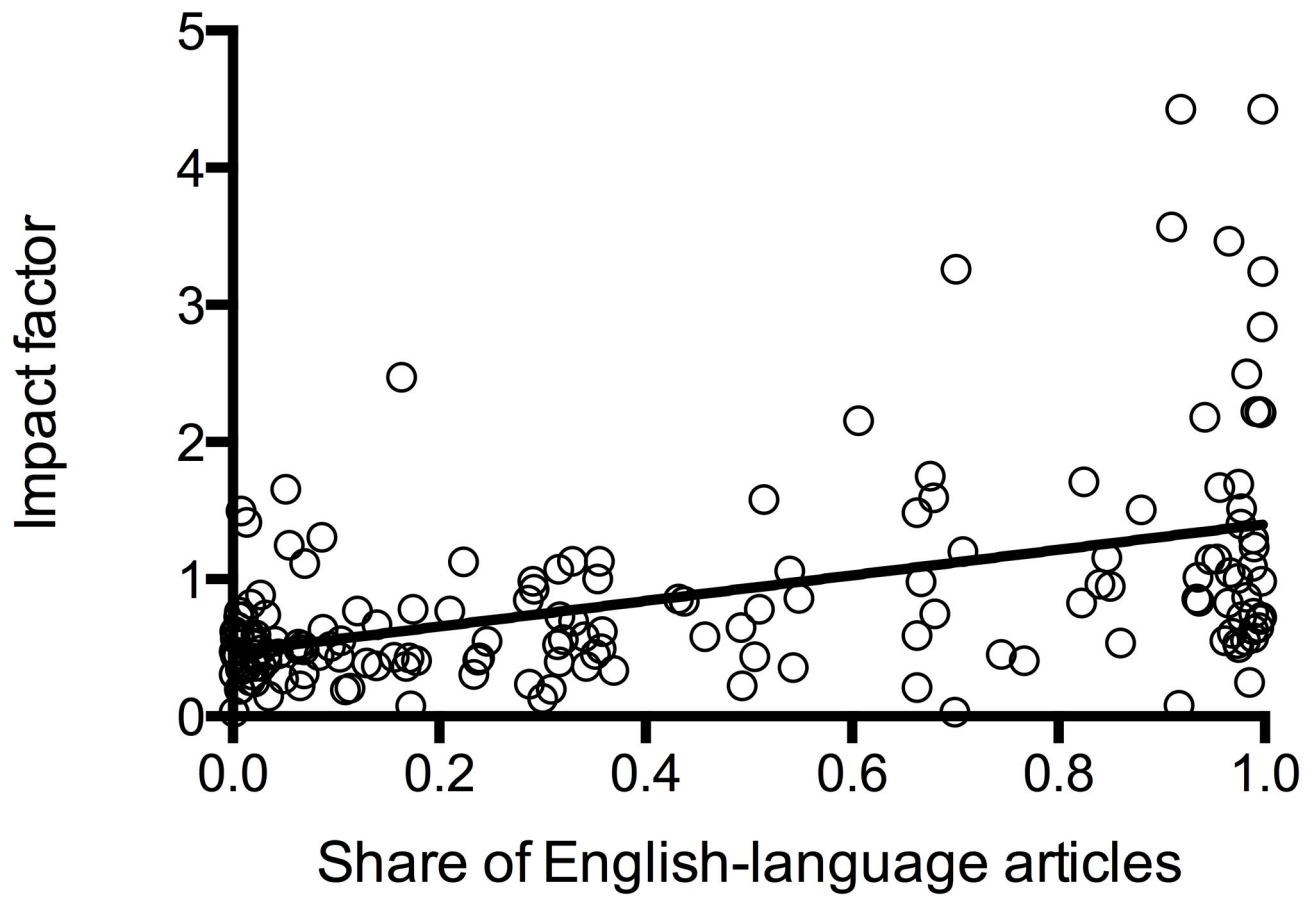
Overall IF in relation to the share of English-language articles. There was also a positive correlation with a weaker Pearson r (r = 0.47, p = <0.0001).

**Figure 4:**
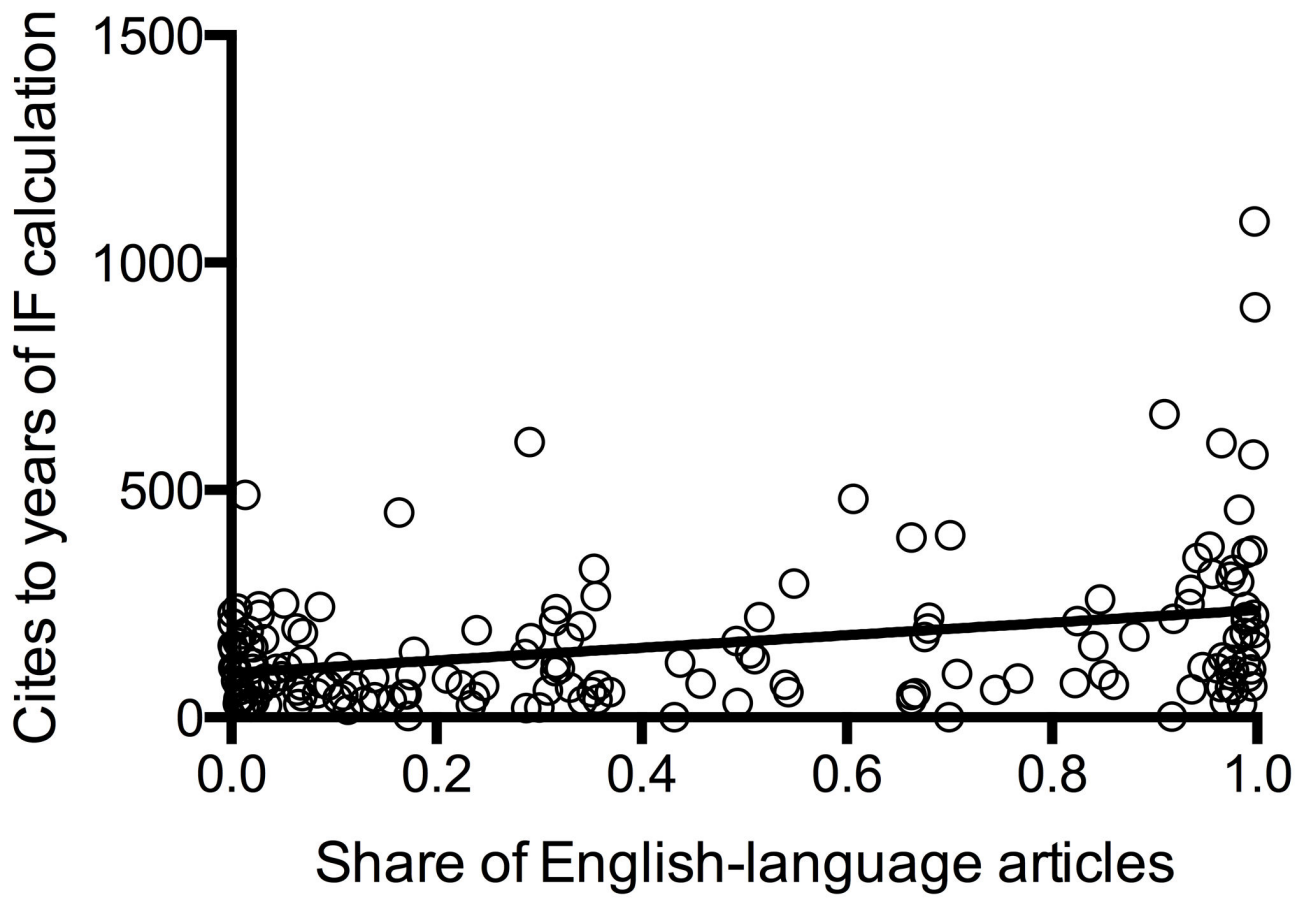
Cites to years of IF calculation in relation to the share of English-language articles. Even the total number of all cites cites showed an association with the share of English-language articles (r = 0.34, p = <0.0001).

**Figure 5:**
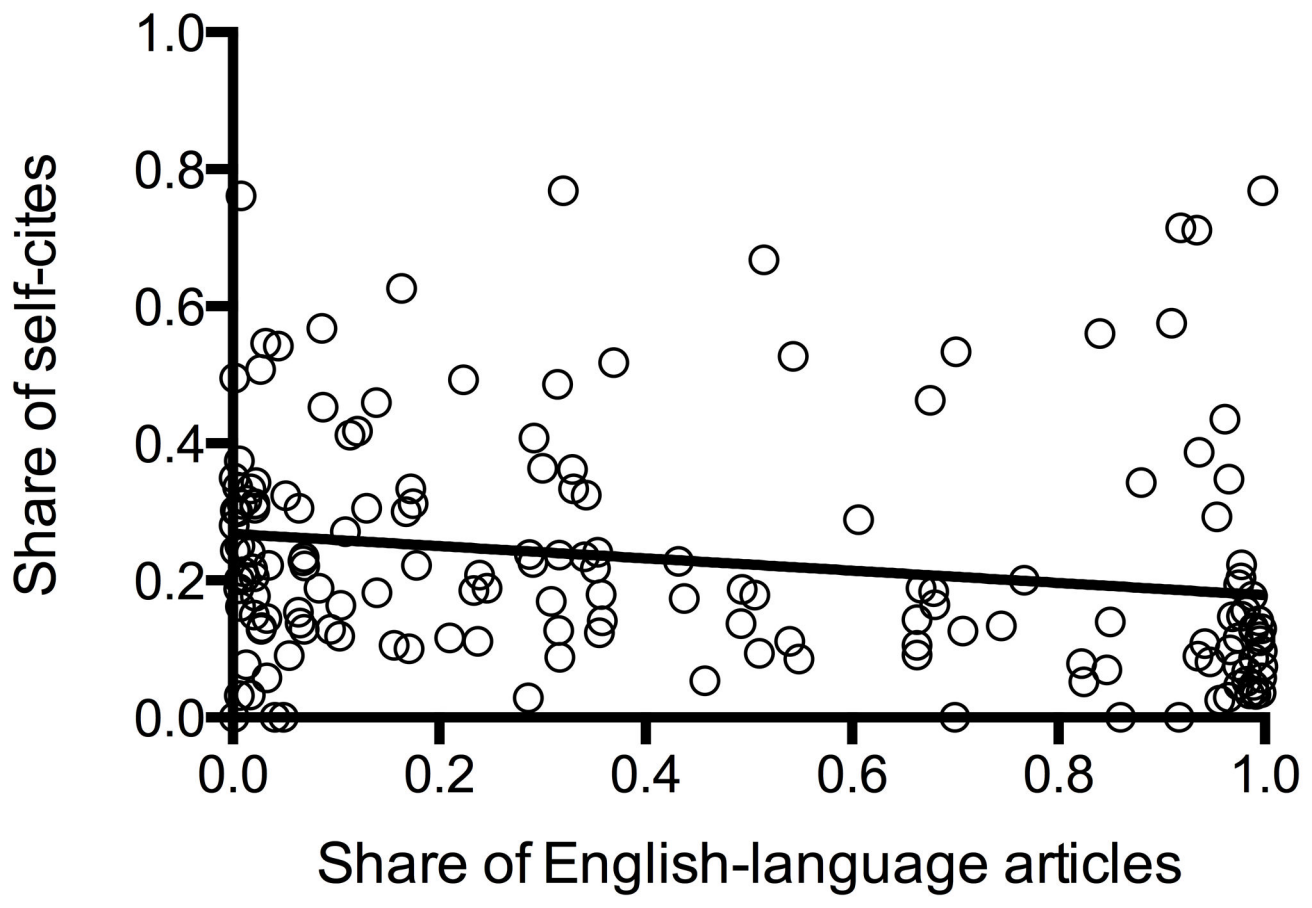
Share of self-citations in relation to the share of English-language articles. There was a weak but negative correlation: the greater the share of English-language articles, the fewer self-citations (r = -0.2, p = 0.009).

We found a significant, though moderate, positive correlation of the share of English-language articles in the years of interest with the IF calculated without self-citations (Pearson r=0.56, p = <0.0001), a weaker correlation with the overall IF (Pearson r = 0.47, p = <0.0001), and the cites to years of IF calculation (Pearson r = 0.34, p = <0.0001) and a weak negative correlation with the share of self-citations (Pearson r = -0.2, p = 0.009) (see Figures **2**-**5**). We also found a significant correlation of the share of self-citations and the share of English articles on the IF without self-citations: the less self-citations and the more English articles the higher the IF without self-citations (see Table **2**). The IF without self-citations was also associated with the journal‘s country of origin as shown in Table **3**: North American journals had a higher IF when calculated without self-citations. 

**Table 2:** Correlation of the share of English-language articles, share of self-cites and the number of cites to years used in IF calculation to the IF without self-citations; the share of English-language articles had a moderate positive influence, whereas the share of self-citations exerts a negative one: The more English articles and the less self-citations the higher the IF without self-citations.

	**Regression coefficient B**	**Standard error**	**p**
**Share of English-language articles**	0.360	0.065	<0.001
**Share of self-cites**	-0.919	0.231	<0.001
**Cites to years used in IF calculation**	0.002	<0.001	<0.001

However, the number of cites to years used in IF calculation seems to have less influence.

**Table 3:** IF without self-cites in other territory groups compared to Northern and Western Europe; the average IF without self-cites of North American journals was 0.577 greater than the IF of Northern and Western European journals, lower in other territory groups.

	**Number**	**Average IF without self-cites**	**Difference between IF without self-cites in other territories and in Northern and Western Europe**	Standard**error**	**p**
**Northern and Western Europe**	79	0.699	0	0.053	-
**Southern and Eastern Europe**	40	0.456	-0.243	0.091	0.008
**North America**	15	1.276	+0.577	0.131	<0.001
**Middle and South America**	27	0.446	-0.253	0.104	0.016
**Asia and Africa**	7	0.419	-0.28	0.183	0.129

The results were similar for the overall IF.

## Discussion

Our analysis of 168 medical multi-language journals has revealed a significant association of the share of original English-language publications in a journal with the proportion of self-citations and its IF: the more English-language articles, the smaller the share of self-citations and the higher the IF. This finding suggests that a greater share of articles in English improves international recognition and therefore fewer self-citations are needed to artifactually increase the IF. On the other hand, this may be caused by authors citing articles in their mother tongue when writing articles in the same language. However, when preparing an English article an author would rather not cite articles written in another language. We also observed an influence of journals‘ countries of origin and the languages used besides English on the results: journals originating in North America tend to have a higher IF, followed by journals from Northern and Western Europe. However, European journals may catch up with America in the future [7]. Most likely, our findings can be ascribed to the number of potential readers that depend on the article language, the country of origin, and the historically developed structures and reputation of medical societies and journals. Therefore, it might be useful for the editors of a journal from a small country to know that articles in English can enhance their journal‘s international visibility and IF. However, journals may aim a better IF by increasing the share of self-citations but that has no influence on the number of potential readers and the possible international recognition of a journal. This is especially important for small national journals that might lose IF in comparison to top-ranked journals [8]. 

The advantages and disadvantages of using the IF as a measure of a journal’s recognition and quality are controversially discussed in the medical literature [9-14]. Basically, the IF is the frequency of citations to the average number of articles published in a scientific journal in a specific period of time, for example the year 2010. The actual impact factor, therefore, is calculated by dividing the total number of an indexed journal’s current year citations to articles in the journal published in the previous two years by the number of “citable items” (e.g., original research articles, reviews or notes) published by the journal in the same period of time. Recent years have witnessed some studies investigating citation practices and trends in all or specific parts of the medical literature as well as potential influences on the IF. The online status of an article, for example, has been shown to affect the IF [15,16]. Even the length of an article’s title is supposed to have an influence on the number of received citations: the longer the title, the more citations [17]. A possible influence of industry on the IF has been discussed in a recent publication [18].

There might be some other influences on the results that are hard to measure. The quality of a publication may differ between non-English and English articles because high quality studies are potentially more likely to be published by the authors in English because of the increased international recognition. On the other hand, in case of lower quality studies, the same authors might be more likely to choose his native language for publication. The medical category of the journal’s WoS provides (e.g., internal medicine or radiology) is also an interesting parameter affecting IF and citation characteristics. However, 37 of the 168 journals analyzed here are listed in two or more categories. Thus, a clear allocation of all journals and therefore a robust statistical analysis of the influence of category is not possible. Some authors doubt that there is a correlation between the share of English-language articles and IF [19,20]; however, it must be noted that these authors analyzed only a selected journal or journal category. Our findings include the available data of all multi-language medical journals with articles published in 2008 and 2009. 

However, our analysis covers only a short period in time. Further investigations need to be conducted to compare the development of the share of English-language articles in multi-language journals over an extended period with changes in IF and self-citation practices [21]. Such studies might provide a different perspective on the issue of English and native language use in the medical literature. Furthermore, it might be interesting to proceed to a publication-based analysis although the assessment of all 127,852 publications would be an enormous challenge. Unfortunately, only about 30% (216 of 709) of the journals PubMed lists were registered in WoS. For example, there were many Chinese and Russian journals with no available citation data in WoS. Nevertheless, we believe that our sample is adequate for getting a robust overview, at least concerning multi-language journals published in Europe and those published elsewhere in Spanish or Portuguese. The increasing use of English in the medical literature has been criticized repeatedly and harshly [22]. Therefore, it is left to authors and publisher to carefully consider the pros and cons and decide whether or not an English article is suitable for a particular carrier or journal. 

## Conclusion

Publishing academic papers in English in multi-language medical journals increases the international visibility and recognition as measured by the IF. At the same time, more English-language articles lead to a smaller share of self-citations required by multi-language journals. 

## References

[1] Kurmis AP (2003) Understanding the limitations of the journal impact factor. J Bone Joint Surg Am 85: 2449-2454. PubMed: 14668520. 14668520 10.2106/00004623-200312000-00028

[2] Foo JYA (2011) Impact of excessive journal self-citations: A case study on the folia phoniatrica et logopaedica journal. Sci Eng Ethics 17: 65-73. doi:10.1007/s11948-009-9177-7. PubMed: 19798588. 19798588

[3] Tighe P, Rice KJ, Gravenstein N (2011) Artifactual Increase in Journal Self-Citation. Anesth Analg 113: 378-382. doi:10.1213/ANE.0b013e31821d72e5. PubMed: 21596873. 21596873

[4] Mueller PS, Murali NS, Cha SS, Erwin PJ, Ghosh AK (2006) The association between impact factors and language of general internal medicine journals. Swiss Med Wkly 136: 441–443. PubMed: 16862464. 16862464 10.4414/smw.2006.11496

[5] Poomkottayil D, Bornstein MM, Sendi P (2011) Lost in translation: the impact of publication language on citation frequency in the scientific dental literature. Swiss Med Wkly 141: 30 PubMed: 21279857. 10.4414/smw.2011.13148 21279857

[6] Grégoire G, Derderian F, Le Lorier J (1995) Selecting the language of the publications included in a meta-analysis: is there a Tower of Babel bias? J Clin Epidemiol 48: 159-163. doi:10.1016/0895-4356(94)00098-B. PubMed: 7853041. 7853041

[7] Karageorgopoulos DE, Lamnatou V, Sardi TA, Gkegkes ID, Falagas ME (2011) Temporal trends in the impact factor of European versus USA biomedical journals. PLOS ONE 6: e16300. doi:10.1371/journal.pone.0016300. PubMed: 21347409. 21347409 PMC3036587

[8] JYA Foo (2009) A 9-year analysis of bibliographical trends for journals in the subject category of general and internal medicine. Accountability Res 16: 127-152. doi:10.1080/08989620902984080. 20183158

[9] Garfield E (1999) Journal impact factor: a brief review. Canadian Medical Association Journal 161: 979-980. 10551195 PMC1230709

[10] Rieder S, Bruse CS, Michalski CW, Kleeff J, Friess H (2010) The impact factor ranking—a challenge for scientists and publishers. Langenbecks Arch Surg 395: 69-73. doi:10.1007/s00423-010-0623-4. PubMed: 20306337. 20306337

[11] Seglen PO (1997) Why the impact factor of journals should not be used for evaluating research. BMJ 314: 498–502. doi:10.1136/bmj.314.7079.498. PubMed: 9056804. 9056804 PMC2126010

[12] Marcovitch H (2010) Editors, publishers, impact factors, and reprint income. PLoS Med 7: e1000355. 21048987 10.1371/journal.pmed.1000355 PMC2964337

[13] Satya-Murti S (2006) Interpreting the Medical Literature. J Am Med Assoc 296: 1410-1411.

[14] Rizkallah J, Sin DD (2010) Integrative approach to quality assessment of medical journals using impact factor, eigenfactor, and article influence scores. PLOS ONE 5: e10204. doi:10.1371/journal.pone.0010204. PubMed: 20419115. 20419115 PMC2855371

[15] Mueller PS, Murali NS, Cha SS, Erwin PJ, Ghosh AK (2006) The effect of online status on the impact factors of general internal medicine journals. Neth J Med 64: 39-44. PubMed: 16517987. 16517987

[16] Gargouri Y, Hajjem C, Larivière V, Gingras Y, Carr L et al. (2010) Self-selected or mandated, open access increases citation impact for higher quality research. PLOS ONE 5: e13636. doi:10.1371/journal.pone.0013636. PubMed: 20976155. 20976155 PMC2956678

[17] Habibzadeh F, Yadollahie M (2010) Are shorter article titles more attractive for citations? Cross-sectional study of 22 scientific journals. Croat Med J 51: 165-170. doi:10.3325/cmj.2010.51.165. PubMed: 20401960. 20401960 PMC2859422

[18] Lundh A, Barbateskovic M, Hróbjartsson A, Gøtzsche PC (2010) Conflicts of interest at medical journals: the influence of industry-supported randomised trials on journal impact factors and revenue–cohort study. PLOS Med 7: e1000354. 21048986 10.1371/journal.pmed.1000354 PMC2964336

[19] Winkmann G, Schlutius S, Schweim HG (2002) [Citation rates of medical German-language journals in English-language papers--do they correlate with the impact factor, and who cites? (reprint)]. Klin Monatsbl Augenheilkd 219: 72–78. doi:10.1055/s-2002-23505. PubMed: 11932815. 11932815

[20] Ruano-Ravina A, Álvarez-Dardet C (2012) Evidence-based editing: factors influencing the number of citations in a national journal. Ann Epidemiol. 10.1016/j.annepidem.2012.06.104 22819434

[21] Wang J (2013) Citation time window choice for research impact evaluation. Scientometrics 94: 851-872. doi:10.1007/s11192-012-0775-9.

[22] Haße W, Fischer RJ (2010) Citation characteristics of German authors in "Der Chirurg": hegemony of the impact factor. Chirurg 81: 361-364. doi:10.1007/s00104-009-1786-9. PubMed: 19760378. 19760378

